# Multi-resolution terrestrial hyperspectral dataset for spectral unmixing problems

**DOI:** 10.1016/j.dib.2022.108331

**Published:** 2022-05-29

**Authors:** C.V.S.S. Manohar Kumar, Sudhanshu Shekhar Jha, Rama Rao Nidamanuri, Vinay Kumar Dadhwal

**Affiliations:** a Department of Earth and Space Sciences, Indian Institute of Space Science and Technology, Valiamala, Thiruvananthapuram, Kerala, India; b National Institute of Advanced Studies, Bengaluru, India

**Keywords:** Hyperspectral remote sensing, Spectral unmixing, Spectroradiometer, Mixture modelling, Target detection, PoC, proof-of-the-concept, BFM, background-free-mixture, SBM, single-background-mixture, MBM, multiple-background-mixture, THI, terrestrial hyperspectral imager

## Abstract

Recent developments in the miniaturization of hyperspectral imaging sensors have given rise to the increased use of hyperspectral imagery as the primary data for evaluating spectral unmixing algorithms in applications such as industrial quality control, agriculture, mineral mapping, military, etc. This article presents an ultra-high-resolution hyperspectral imagery dataset for undertaking benchmark studies on spectral unmixing. A terrestrial hyperspectral imager (THI) is used for imaging the target scene with the camera sensor pointing horizontally towards the target scene. The datasets are acquired at various spatial resolutions ranging from 1 mm to 2 cm. The targeted scene contains several paper-based panels, each size of 2 cm x 2 cm and filled with different colours and proportions, glued to a black background board that maintains a distinguishable distance between one another. In addition to the hyperspectral imagery data acquisitions, reference spectral signatures of the candidate mixture materials are obtained by in-situ hyperspectral reflectance measurements using a spectroradiometer. The hyperspectral image acquisition and the in-situ spectral signatures of the target scene are collected under natural illumination conditions. The proposed datasets are designed for undertaking proof-of-the-concept (PoC) studies in spectral unmixing. The datasets are also valuable for evaluating the performance of different statistical and machine learning algorithms for target detection, classification, and sub-pixel classification algorithms.

**Specifications Table**

**Table:** 

Subject	Engineering (General)
Specific subject area	Remote Sensing - Hyperspectral Remote Sensing, Spectral Unmixing, Spectral Mixture Analysis
Type of data	Hyperspectral imagery (Raster) Comma Separated Value Excel file
How data were acquired	• Hyperspectral images were acquired using a push-broom hyperspectral imaging system (Make and Model: Headwall Photonics Inc. USA; VNIR A) • In-situ spectral measurements were acquired using a field spectroradiometer (Make and Model: Spectra Vista Corporation, USA; HR-1024i)
Data format	• Raw/processed hyperspectral imagery datasets are in ENVI .hdr file format • Raw in-situ spectra are saved in ENVI .hdr file format • Processed in situ spectra are saved in .xlsx file format
Description of data collection	• Five paper-material of different colours and black background were chosen as the candidate materials for acquiring imagery • The colour proportions were arranged in 2 cm X 2 cm in different ways • A ground-based hyperspectral imaging sensor was used for imagery acquisition • Spectral library based on in-situ reflectance spectra collected using a field spectroradiometer
Data source location	An experimental setup was laid out at the Indian Institute of Space Science and Technology, Thiruvananthapuram, India Geographical location: 8°37.482′N 77°1.964′E City/Town/Region: Valiamala, Thiruvananthapuram, Kerala Country: India
Data accessibility	Repository name: Mendeley Data Data identification number: http://dx.doi.org/10.17632/pts6cw4xbt.1 Direct URL to data: https://data.mendeley.com/datasets/pts6cw4xbt/1
Related research article	Manohar Kumar, C. V. S. S., Jha, S. S., Nidamanuri, R. R. and Dadhwal, V. K. Benchmark studies on pixel-level spectral unmixing of multi-resolution hyperspectral imagery. International Journal of Remote Sensing, 2022. 43(4), 1451-1484. DOI: https://doi.org/10.1080/01431161.2022.2040755

**Value of the Data**

• Spectral unmixing has been one of the classical approaches of analysing remote sensing imagery at the sub-pixel level. Initially developed as an analytical technique for estimating fractional abundances of surface materials in coarse resolution multispectral remote sensing, spectral unmixing has been extensively studied during the last three decades [1], [2], [3], [4], [5]. The advances in hyperspectral imaging technology have the potential to expand the application of spectral unmixing in various precision-material fractions retrievals in military, mineral mapping, food processing, quality control etc. Due to the lack of pixel-to-pixel ground reference measurements, the various methods and algorithms designed for spectral mixture modelling are often faced with generic or synthetic datasets to model and validate the results [6,7]. In addition, the availability of benchmark datasets to assess the impact of varying spatial resolution for sub-pixel classification is scarce.
• The problem of spectral unmixing is being pursued using several linear and non-linear approaches. This dataset will be handy for carrying out proof-of-the-concepts (PoC), mathematical constructs, and the performance of the developed processes/algorithms.
• It has often been found that theoretical algorithms have certain limitations when deployed in realistic environments at different spatial scales. As the dataset allows implementing algorithms at the different spatial resolutions of hyperspectral imagery, the dataset will enable the development of scale-invariant methods for spectral unmixing problems.
• The datasets are a valuable resource for researchers and scientists developing algorithms and concepts in solving spectral unmixing, classification, and target detection problems.

## 1 Data Description

The entire data is gathered in a single folder, ‘*Hyperspectral_Unmixing_Data*’ and then zipped. On extracting this zip file, there are two folders, ‘*Hyperspectral_Image_Data*’ and ‘*Spectral_Library*’. ‘*Hyperspectral_Image_Data*’ has two subfolders - ‘*Raw_Data*’ and ‘*Processed_Data*’. In the ‘*Raw_Data*’ folder, there are five radiance files in the standard ENVI “.hdr” format. The ‘*Processed_Data*’ folder is further divided into five subfolders (named 5m, 10m, 25m, 50m, and 62m), which is further divided into three subfolders (named BFM, SBM, and MBM). Each subfolder- BFM, SBM, and MBM- contains ten reflectance files in ENVI “.hdr” file format with 743 spectral bands each.

The file name of each reflectance is represented as “**C_D**m**_X_N**p.hdr”, where capital letters in bold can be replaced by different letters and numbers.

“**C**” – indicates the mixture category by three letters M – Background free mixture (BFM), S – Single background mixture (SBM) and D – Multiple background mixture (MBM).

“**D**” – represents the distance between the sensor and target 5 m, 10 m, 25 m, 50 m and 62 m.

“**X**” – specifies the mixture composition as mentioned in Tables 1 – 3 ranging from 1 to 10.

“**N**” – consists of the number of pixels in a row or column ranging from 16, 10, 8, 5, and 4 w.r.t distance.

‘*Spectral_Library*’ folder is subdivided into two; the first one is “*Raw*” which contains raw reflectance spectral library in the “.hdr” and “.ascii” file formats. In the second “*Processed*” folder, reflectance resampled to the hyperspectral image file in the “.xlsx” file format is provided.

**Fig. 1:**
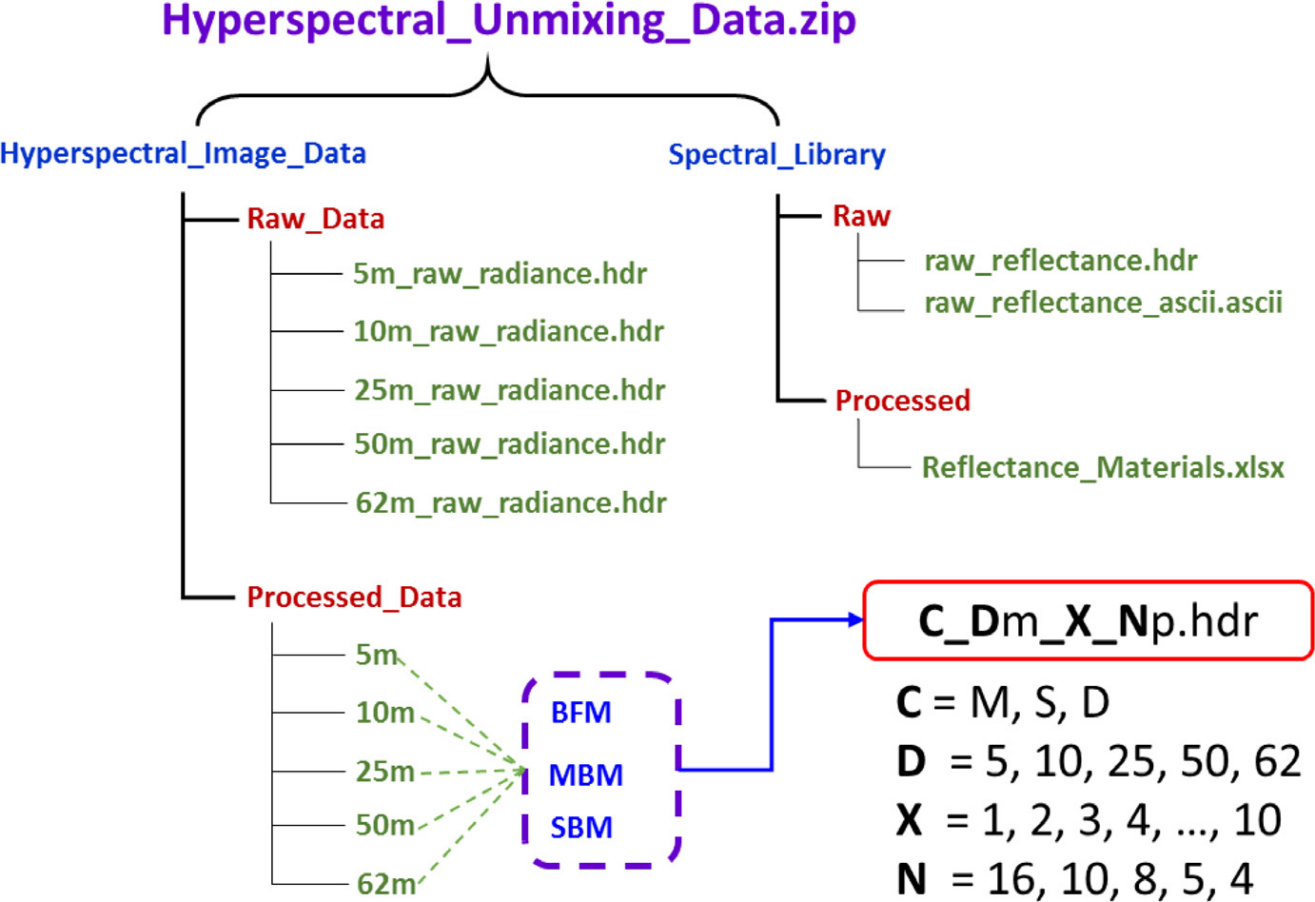
The raw and processed files organization structure of hyperspectral image datasets and the spectral library of in situ reflectance measurements.

The entire hierarchy of this section is shown in Fig. 1.

## 2 Experimental Design

The experiment design is based on the proof-of-the-concept for the assumptions in spectral unmixing as referred in [8]. In this experiment, each grid is of the area of 2 cm x 2 cm and is filled with five distinct colour materials. Each colour-grid represents a distinct material, and hence its reflectance spectrum is treated as an endmember. Various scenarios of mixture combinations are prepared in different sizes, orientation, and spatial distribution of materials with delineable boundaries in a grid. These grids can be seen in realistic form by printing colours in the print grid form. Single colour candidates are treated as pure pixels, and the combination of different colours in a grid represents mixed pixels.

**Fig. 2:**
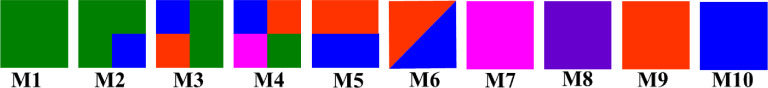
**Reference imagery design set up for background free mixture (BFM) of materials** (Adapted from C. V. S. S. Manohar Kumar, S. S. Jha, R. R. Nidamanuri, V. K. Dadhwal, Benchmark studies on pixel-level spectral unmixing of multi-resolution hyperspectral imagery. International Journal of Remote Sensing, 43 (2022) 1451-1484. doi: 10.1080/01431161.2022.2040755).

We designed three different categories of spectral mixtures using five different colour materials - green, magenta, red, blue, and violent. The first category has ten candidate images – five images representing pure materials and five images representing a mixture of materials with combinations of variable size and spatial orientation inside a grid. In this category, there is no local background in the imagery and presents only pure and mixtures of candidate materials spectra. For ease of reference, this category of images is given the name ‘background-free-mixture (BFM)’. The composition of mixtures is shown in Fig. 2 and its areal proportion details are presented in Table 1.

**Table 1:** **Background free mixture (BFM): Label and areal proportion (%)** (Adapted from C. V. S. S. Manohar Kumar, S. S. Jha, R. R. Nidamanuri, V. K. Dadhwal, Benchmark studies on pixel-level spectral unmixing of multi-resolution hyperspectral imagery. International Journal of Remote Sensing, 43 (2022) 1451-1484. doi: 10.1080/01431161.2022.2040755).

Mixture	Green	Magenta	Red	Blue	Violet
M1	100	0	0	0	0
M2	75	0	0	25	0
M3	50	0	25	25	0
M4	25	25	25	25	0
M5	0	0	50	50	0
M6	0	0	50	50	0
M7	0	100	0	0	0
M8	0	0	0	0	100
M9	0	0	100	0	0
M10	0	0	0	100	0

**Fig. 3:**
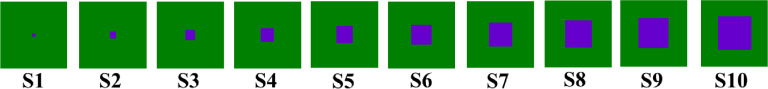
**Design of imagery acquisition for materials with a single background mixture (SBM)** (Adapted from C. V. S. S. Manohar Kumar, S. S. Jha, R. R. Nidamanuri, V. K. Dadhwal, Benchmark studies on pixel-level spectral unmixing of multi-resolution hyperspectral imagery. International Journal of Remote Sensing, 43 (2022) 1451-1484. doi: 10.1080/01431161.2022.2040755).

The second category has ten images representing different mixtures of two different materials indicated by two different colours. Each candidate mixture consists of different proportions of green colour behaving as local background, and violet colour material as foreground. This set is termed as ‘single-background-mixture (SBM)’. The areal distributions of the two endmembers are listed in Table 2, and the visualization of the dataset is shown in Fig. 3.

**Table 2:** **Same background mixture (SBM): Label and areal proportion (%)** (Adapted from C. V. S. S. Manohar Kumar, S. S. Jha, R. R. Nidamanuri, V. K. Dadhwal, Benchmark studies on pixel-level spectral unmixing of multi-resolution hyperspectral imagery. International Journal of Remote Sensing, 43 (2022) 1451-1484. doi: 10.1080/01431161.2022.2040755).

Mixture	Green (Background)	Violet (Foreground)
S1	99.75	0.25
S2	99	1
S3	97.75	2.25
S4	96	4
S5	93.75	6.25
S6	91	9
S7	87.75	12.25
S8	84	16
S9	79.75	20.25
S10	75	25

**Fig. 4:**
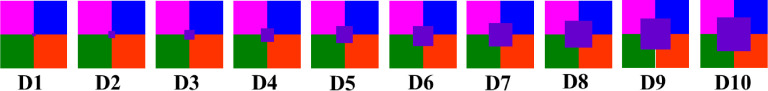
**Design of imaging set up for materials with multiple background mixture (MBM)** (Adapted from C. V. S. S. Manohar Kumar, S. S. Jha, R. R. Nidamanuri, V. K. Dadhwal, Benchmark studies on pixel-level spectral unmixing of multi-resolution hyperspectral imagery. International Journal of Remote Sensing, 43 (2022) 1451-1484. doi: 10.1080/01431161.2022.2040755).

The third category, labelled as ‘multiple-background-mixture (MBM)’, has a background of four different materials with fixed areal proportions. The foreground material (violet colour) has different proportions indicating different levels of interactions of background and foreground materials. Fig. 4 represents the combination candidate mixture set, and its areal distributions are listed in Table 3.

**Table 3:** **Multiple background mixture (MBM): Label and areal proportion (%)** (Adapted from C. V. S. S. Manohar Kumar, S. S. Jha, R. R. Nidamanuri, V. K. Dadhwal, Benchmark studies on pixel-level spectral unmixing of multi-resolution hyperspectral imagery. International Journal of Remote Sensing, 43 (2022) 1451-1484. doi: 10.1080/01431161.2022.2040755).

	Background				Foreground
Mixture	Green	Magenta	Red	Blue	Violet
D1	24.9375	24.9375	24.9375	24.9375	0.25
D2	24.75	24.75	24.75	24.75	1
D3	24.4375	24.4375	24.4375	24.4375	2.25
D4	24	24	24	24	4
D5	23.4375	23.4375	23.4375	23.4375	6.25
D6	22.75	22.75	22.75	22.75	9
D7	21.9375	21.9375	21.9375	21.9375	12.25
D8	21	21	21	21	16
D9	19.9375	19.9375	19.9375	19.9375	20.25
D10	18.75	18.75	18.75	18.75	25

All the three categories of images representing different mixtures were realized by printing the respective colour grids on a standard printing paper and affixed to a wooden board coated in black colour material. A radial distance of 10 cm was maintained to separate each candidate mixture. The black colour behaves as global background to all candidate mixtures. This candidate-mixture material configured wooden board was held onto a tripod to measure by a hyperspectral imaging camera.

## 3 Data Acquisition

**Fig. 5:**
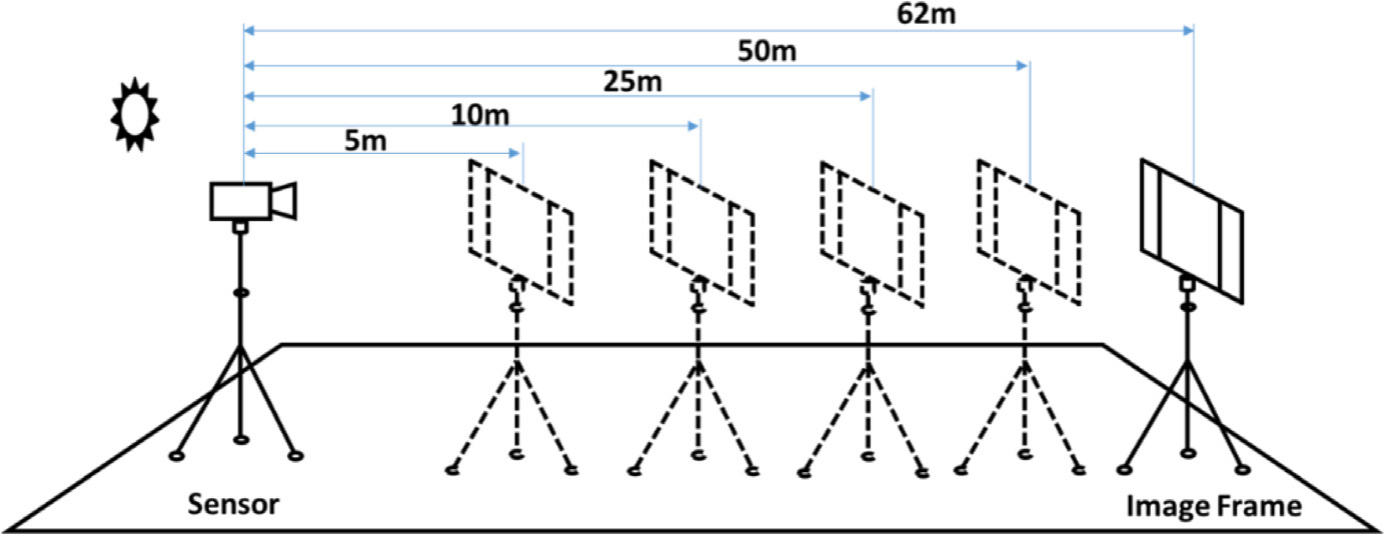
**Synoptic view of the data acquisition set-up: sensor - scene positioning** (Adapted from C. V. S. S. Manohar Kumar, S. S. Jha, R. R. Nidamanuri, V. K. Dadhwal, Benchmark studies on pixel-level spectral unmixing of multi-resolution hyperspectral imagery. International Journal of Remote Sensing, 43 (2022) 1451-1484. doi: 10.1080/01431161.2022.2040755).

The data acquisition set-up designed at multiple resolutions is shown in Fig. 5. The hyperspectral imagery data was acquired using a terrestrial hyperspectral imager (THI) (Headwall Photonics Inc., USA; Model: A-Series). The spectral imager used in the acquisition has 854 spectral bands in the visible and infrared range of 400 to1000 nm of the electromagnetic spectrum. The sensor uses push-broom imaging mode and can record 1004 pixels in a single column.

An additional rotating stage was attached between the sensor and tripod to scan an area. The speed of the rotational stage (angular view range 0° – 360°) was manually adjusted between 0.01° to 30° according to the framerate of the sensor. While acquiring, the signal saturation was avoided by manually adjusting the exposure time. The exposure time was calculated by focusing on a white reference plate (prepared by barium sulphate material) until the black and white lines are separable on the connected computer live feed screen. Based on the exposure time the rotating speed was calculated and given the input through the rotating stage control unit attached to the same computer.

**Fig. 6:**
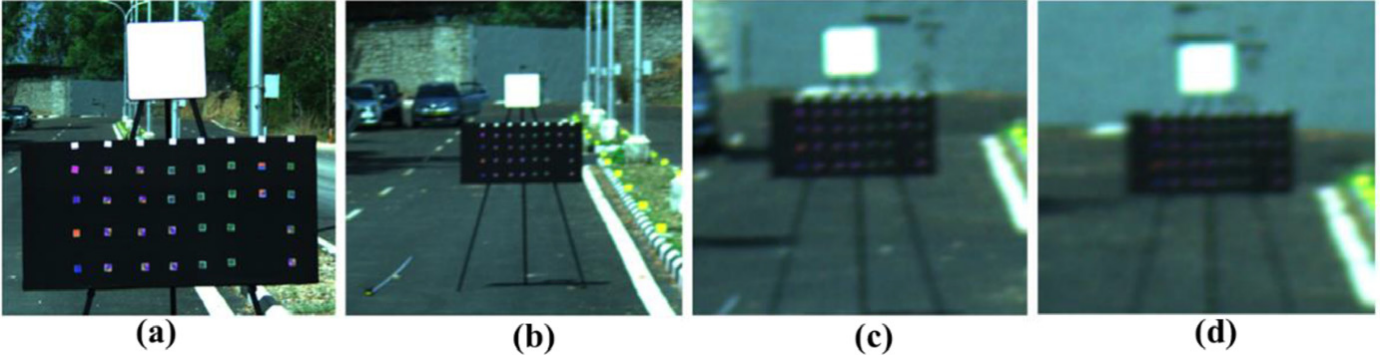
The targeted scene at different distances (a: 5m, b: 25m, c: 50m, and d: 62m) from the hyperspectral imager.

The effective spatial resolution depends on the lens used and the distance between the targeted scene and the sensor. We acquired multiple datasets of hyperspectral imagery at various spatial resolutions ranging from 1 mm (5 m distance between sensor and scene) to 2 cm (62 m distance between sensor and scene) using a 23 mm lens. The actual targeted image frame used in the field for acquisition is shown in Fig. 6.

The in-situ measurements were collected using a high resolution spectroradiometer (HR-1024i, Spectra Vista Corporation, USA). This instrument records reflected the radiance of the target material in the 350 – 2500 nm portion of the electromagnetic spectrum.

The hyperspectral image acquisition and in situ spectral measurements were acquired on January 31, 2019, from 11:00 to 13:00 Hrs in IST local time. The procedure suggested by [9] was adapted for acquiring the reflectance measurements.

## 4 Data Pre-processing

The hyperspectral imagery datasets acquired are in radiance units. In the first stage, the radiance data were converted into reflectance data through the reference spectral imagery acquired over a white reference plate of known spectral calibration. The reflectance data were further smoothed in the spectral dimension using the Savitzky-Golay filter [10]. After the filtering, the data were spectrally subset to confine to 400 – 920 nm range to eliminate uncalibrated extremely noisy bands. The image data were spatially cropped to carve out several subsets based on the mixture categories (BFM, SBM, and MBM). At different spatial resolutions indicated by the distance from the sensor, the number of distinct pixels in each subset representing different material proportions is listed (Table 4).

**Table 4:** Actual mixture to number of pixels.

Distance(m)	No. of pixels	Total (%)	2 × 2 cm^2^ (%)	Black-background (%)
5	16 × 16	100	60.3771	39.6228
10	10 × 10	100	38.6413	61.3586
25	8 × 8	100	9.6603	90.3396
50	5 × 5	100	6.1826	93.8173
62	4 × 4	100	6.2827	93.7172

**Fig. 7:**
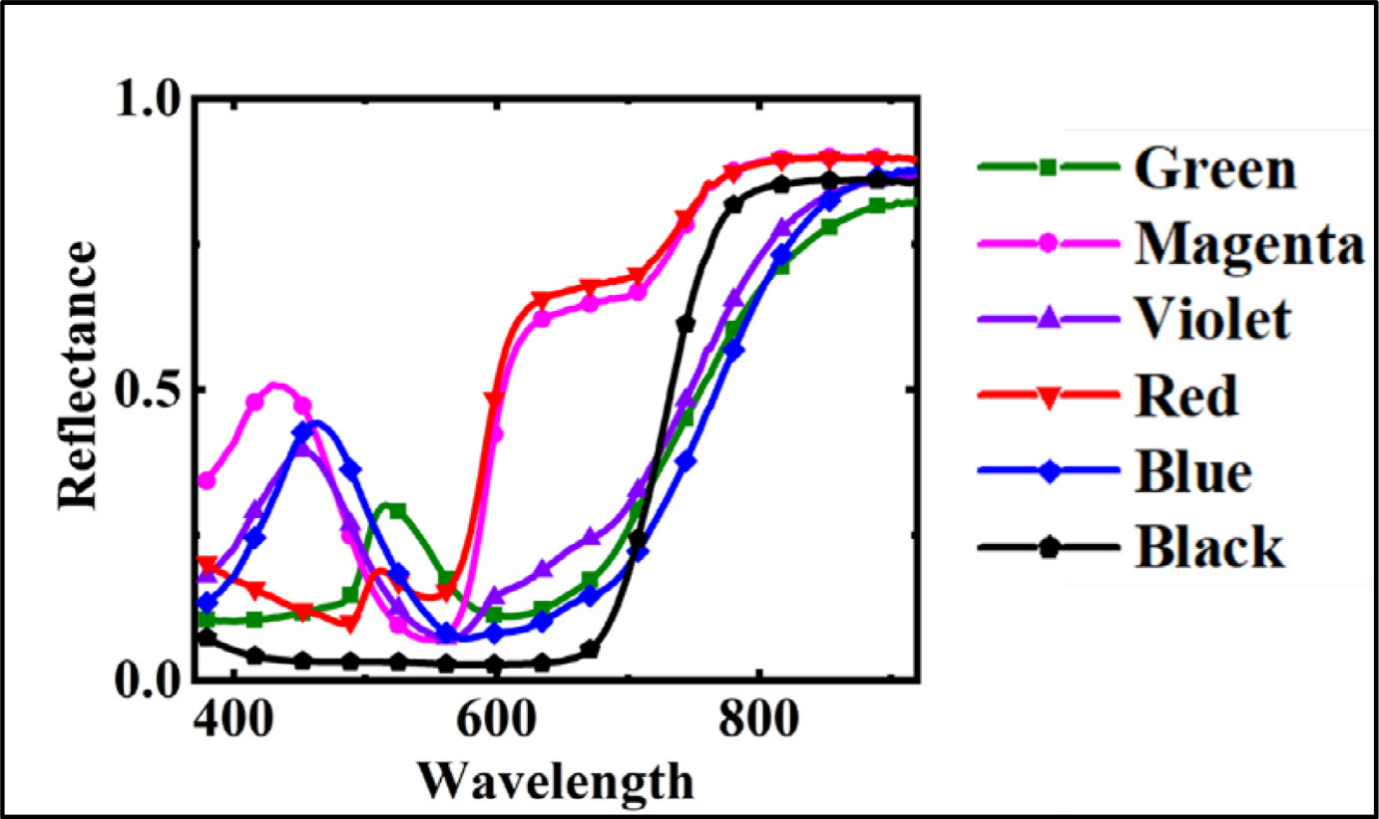
**The pre-processed spectral profiles of different colour materials acquired from in-situ spectral measurements** (Adapted from C. V. S. S. Manohar Kumar, S. S. Jha, R. R. Nidamanuri, V. K. Dadhwal, Benchmark studies on pixel-level spectral unmixing of multi-resolution hyperspectral imagery. International Journal of Remote Sensing, 43 (2022) 1451-1484. doi: 10.1080/01431161.2022.2040755).

The reflectance spectral measurements acquired using the field spectroradiometer were organized in the form of a spectral library. This spectral library was resampled to conform to the datasets of hyperspectral imagery using spectral response function modelling. The different colour material's spectral signatures are shown in Fig. 7.

## Ethics Statements

No animal or human subjects are used in the experimental set-up. The data is not collected from any social media platform.

## CRediT Author Statement

**Manohar Kumar C. V. S. S.:** data acquisition and pre-processing, writing original draft; **Sudhanshu Shekhar Jha:** data acquisition, editing and review; **Rama Rao Nidamanuri:** conceptualization, experiment design, review; **Vinay Kumar Dadhwal:** data quality review, supervision.

## Declaration of Competing Interest

The authors declare that they have no known competing financial interests or personal relationships which have, or could be perceived to have, influenced the work reported in this article.

## Data Availability

Multi-resolution Terrestrial Hyperspectral Dataset for Spectral Unmixing Problems (Original data) (Mendeley Data).
